# Economic expectations and anxiety during the COVID-19 pandemic: a one-year longitudinal evaluation on Italian university students

**DOI:** 10.1007/s11135-022-01330-y

**Published:** 2022-02-28

**Authors:** Giovanni Busetta, Maria Gabriella Campolo, Demetrio Panarello

**Affiliations:** 1 Department of Economics, University of Messina, Via dei Verdi 75, 98122 Messina, Italy; 2 Department of Statistical Sciences “Paolo Fortunati”, University of Bologna, Via delle Belle Arti 41, 40126 Bologna, Italy

**Keywords:** Coronavirus outbreak, Economic prospects and uncertainty, Mental health, Psychological well-being, State and trait anxiety, A12, I18, I23, I31, P46

## Abstract

The COVID-19 pandemic has produced an extensive aggravation of people’s anxiety level. Different policies aimed at fighting the spread of the virus could affect anxiety in various ways. We built an ad hoc web-based survey, administered to the student population of three Italian universities at the beginning of the pandemic and at one year’s distance, to collect information on retrospective and current anxiety levels and the underlining reasons. The survey also included questions concerning sociodemographic, economic, labor, lifestyle, academic career, and on-line teaching features, which prevents students from identifying the main survey topic to be anxiety. This research aims at assessing the change in anxiety levels between the analyzed periods and the main determinants of such change, focusing on students’ economic expectancies. Results from a Poisson regression model show that anxiety has increased compared to both the pre-pandemic level and the one quantified during the first lockdown. This increase is revealed to be mostly driven by economic and career-related uncertainties, rather than by job loss and proximity to COVID-19. Thus, policymakers should take action to provide certainties both in terms of economic prospects and reopening strategies, especially to avoid that the resulting increase in anxiety translates into an amplified suicide risk.

## Introduction

Coronavirus SARS-CoV-2 was first discovered in December 2019 in Wuhan, China. Since then, it rapidly spread through most of the countries worldwide. Deeply, the contagion has reached Italy one month later (January 30th, 2020), due to two COVID-19 positive cases detected in Chinese tourists visiting Italy. The number of confirmed cases increased on February 21st, 2020, when sixteen people in Lombardy and Veneto were confirmed to be infected. Several towns in Lombardy were rapidly placed on lockdown in the following days, due to a large number of infections. Italy implemented a nationwide lockdown on March 10th, 2020, which lasted until May 3rd, letting the whole country become a “protected area”.

In that period, residents were permitted to leave their homes only to buy food and medicines, while attending school or performing non-essential jobs was not allowed (Borri et al., 2021; Panarello and Tassinari, 2022). Moreover, any kind of public gatherings and public transport were curtailed.

On May 4th, 2020, the so-called “phase 2” began, with a gradual relaxation of the previous containment measures to mitigate the collateral effects of a severe lockdown, as the epidemic curve was in its descent phase. The strategy was to use alternative public health measures such as imposing social distancing and school closures, as well as eliminating mass events, to keep the number of infections under control, while giving people more freedom. Despite this, essential economic activities were never shut down and remained fully functional (Di Porto et al., 2022).

From June 15th, 2020, throughout the summer (phase 3), the measures were furtherly relaxed. Due to the increase in infections in the Italian territory, beginning from August 16th, 2020, restrictive measures became more stringent. Since the beginning of pandemic, the strategy of alternating periods of openings and closings of several commercial establishments and schools was adopted (Alfano et al., 2021) as an alternative to a complete lockdown, which produces negative socio-economic and psychological consequences (see Fardin, 2020 for an overview). The main literature on this topic (Jeong et al., 2016; Liu et al., 2012) suggests that psychological impacts of isolation tend to be long-lasting, widespread and substantial, but it suggests in the meanwhile that the alternative of not imposing any isolation could be even worse (Hull, 2005). Indeed, the psychological literature (see Ho et al., 2021 for an overview) suggests stressful periods such as pandemics to be connected to an increase in stress, anxiety and depression (Salari et al., 2020). In the case of the COVID-19, we are still not able to evaluate whether or not such effect will be long-lasting. Nevertheless, empirical evidence shows the existence of widespread emotional distress in Australia (Brindal et al., 2021), China (Qiu et al., 2020; Ran et al., 2020; Wang et al., 2020), India (Mahanty et al., 2021), Italy (Busetta et al., 2021; Fornili et al., 2021; Mazza et al., 2020), Japan (Ueda et al., 2020), Spain (Planchuelo-Gómez et al., 2020), the United Kingdom (Daly et al., 2020; Shen and Bartram, 2021; Shevlin et al., 2020), the United States (Zheng et al., 2021), and 54 countries in a cross-country framework (Büyükkeçeci, 2021) at the beginning of the COVID-19 pandemic.

Even if strict isolation measures connected to the widespread diffusion of COVID-19 already imply deep psychological consequences, we were wondering whether this opening/closing alternation, which produces collateral insecurity effects, could induce an even deeper negative impact in terms of anxiety.

Indeed, the continuous spread of the pandemic, strict isolation measures and delays in starting schools, colleges, and universities across the country is expected to influence students’ mental health: uncertainty about the future and ambiguity in communication can significantly affect expectations which, along with emotions such as anxiety, play a relevant role in laying the foundation for a sound post-pandemic snap-back (Codagnone et al., 2021; Massaro et al., 2021). Economic anxiety particularly affects young adults and women, which constitute the highest-risk categories in this respect (Fetzer et al., 2021).

Daniele et al. (2020) administered a survey to eight thousand respondents in four Western European countries, finding that a general deterioration in the levels of institutional trust and support for social welfare spending financed by taxes are mostly driven by the COVID-19-driven economic insecurity rather than the pandemic’s health dimension.

Regarding age, young adults (aged 18–30 years) and elderly (older than 60 years) are the two categories that exhibit higher levels of anxiety as an effect of distress factors deriving from pandemic outbreaks (e.g., Ho et al., 2020; Koch and Park, 2022; Wang et al., 2020). This result, referred to young age, is confirmed by studies specifically performed in Italy (Busetta et al., 2021; Mazza et al., 2020) and it is most probably associated with the greater access of young individuals to information and news through social media, which could induce an increase in their stress level.

According to the main literature (see Salari et al., 2020 for an overview), greater levels of anxiety, depression, and stress commonly affect people with higher levels of education, making university students a particularly relevant sample to evaluate the impact of the current pandemic on anxiety. Therefore, this study focuses on the psychological impact of the COVID-19 outbreak among the students of three Italian Universities, respectively located in the South, Center and North of Italy. Specifically, we want to examine how and why anxiety levels change throughout the pandemic, testing the following hypotheses:

- H1: State anxiety in 2021 is higher than trait anxiety and than state anxiety in 2020.
- H2: Differences in the development of state anxiety can be observed depending on students’ level of anxiety pre-pandemic and at the beginning of it.
- H3: Students’ economic expectancies can affect anxiety levels.
- H4: Women are more likely to develop a high level of state anxiety compared to men.

Most previous analyses are affected by methodological limitations, including cross-sectional design and the absence of a control group. Involving an appropriate control group is quite complicated to do because most of the lockdowns took place at a national level at the same time (Prati and Mancini, 2021). As stated by Meda and Slongo (2020), it is dangerous to infer conclusions on the psychological consequences of the COVID-19 pandemic using cross-sectional data which are not supported by a control group. A longitudinal analysis is the other possibility to solve this problem, as it can provide a baseline of pre-pandemic data that allows assessing whether the increase in anxiety is strictly COVID-19-related. However, such a procedure is difficult to perform due to the limited time passed since the onset of the pandemic. In this respect, following the main literature (see Prati and Mancini, 2021 for a review), “longitudinal studies that examine within-person change before and after lockdowns provide important information on the psychological impact of lockdowns”.

Rogers et al. (2020) found a small but significant effect of COVID-19 lockdowns on mental health, most probably because they were not able to consider the effects of long enough pandemic lockdown policies, as we instead do. In particular, the idea is that lockdowns impact selectively and modestly on mental health with no effects on the positive functioning of individuals (Keyes, 2005).

We overcame both problems by collecting a longitudinal dataset and using the State-Trait Anxiety Inventory (STAI). This way, we consider both trait anxiety, as a measure of anxiety captured regardless of the pandemic, and state anxiety, collected from the same individuals in two different moments in the course of the pandemic: at the beginning of it and after one year. The STAI test is composed of two subscales: The State Anxiety Scale (S‐Anxiety), which evaluates the current state of anxiety, and the Trait Anxiety Scale (T‐Anxiety), which evaluates a stable measure of anxiety. Some authors (Romeo et al., 2021) used the same psychological test to assess University students’ level of anxiety, but in that case, they only used state anxiety without controlling for a previous level of anxiety (the Trait Anxiety Scale).

Using both scales and measuring them in two different moments, we can consider: the habitual level of anxiety before the pandemic, the level of anxiety during the first stages of the pandemic, and its development one year after. As the Trait Anxiety levels of the two samples collected at waves 1 and 2 are not significantly different, we can infer that the panel is not significantly different from the overall sample.

Our study aims to assess the psychological effects of restrictive measures one year after the beginning of the pandemic, by analyzing the anxiety level of students enrolled in three Italian Universities (University of Messina, Marche Polytechnic University, University of Udine). This topic is particularly relevant because, up to now, no research has been able to analyze more than the short-term impact of lockdown on mental health (Prati and Mancini, 2021). We succeeded in analyzing mid-term effects, assessing the extent to which repeated and prolonged lockdowns and the fear of the virus itself may contribute to anxiety disorders.

Following one of the few other studies using longitudinal data (e.g., Wang et al., 2020), having a family member infected by COVID-19 is not necessarily associated with higher anxiety levels, although individuals assert to be highly concerned by the health of their relatives. These authors assess that neither prolonged lockdowns nor increasing death rates are associated with significant anxiety shifts in China. Following their results, no significant differences in stress, anxiety and depression levels were found between their first and second wave; moreover, longer pandemic lockdown policies are associated with a limited and transient impact on population mental health.

Previous analyses stated that mental health problems did not aggravate massively with lockdowns, but they cannot be taken as evidence that mental problems had not arisen due to the COVID-19 lockdown, since they only analyzed lockdowns’ initial periods. On the contrary, we are particularly worried that longer-lasting lockdowns could increase the level of anxiety of particularly fragile groups of individuals such as students, influencing their learning performances and, through them, their personal equilibrium. Our study took place during a longer period and highlighted that the average level of anxiety has hugely worsened after repeated and prolonged restrictive measures, at least on a particularly sensitive category such as university students.

The results could derive from different factors, related for instance to social isolation and socio-economic components such as financial insecurity and job loss.

The remainder of this paper is organized as follows. Section 2 provides an overview of the literature on the topic; in Sect. 3, we report the characteristics of the survey and the methods used in our analyses; Sect. 4 shows the main results; finally, in Sect. 5, we discuss the implications of our results and provide some concluding remarks.

## Review of the literature

Lockdown consists of restricting people’s movements in order to limit the spread of contagions, by reducing the risk that potentially infected people will infect others. This is one of the public health measures which were applied all over the world during major disease outbreaks. It has been used, in different countries, ways and timings, during the COVID-19 outbreak. Following previous research (e.g., Haider et al., 2020), the lockdown can be defined as an emergency and temporary measure imposed by governmental authorities to oblige the entire population of a city, region or nation to limit social contacts through stay-at-home orders. This technique altered people’s daily lives, way of working, leisure activities and social interactions. In some periods, entire cities and nations were effectively placed under massive isolation.

Direct socio-economic effects of lockdowns usually consist of job losses and business closures (Miles et al., 2020). The COVID-19-related non-pharmaceutical interventions have impacted on the already existing socio-economic inequalities (Davillas and Jones, 2021; Perugini and Vladisavljevic, 2021). Indeed, some jobs are more flexible than others as regards the possibility to be performed remotely: those requiring lower skills or education are usually the most affected by the closures and, thus, vulnerable economic groups such as females (Alon et al., 2020) and young people (Brunori et al., 2021) will be ones suffering the most by the economic risks brought about by COVID-19 and, in particular, the most exposed to the prospect of unemployment. Furthermore, Adams-Prassl et al. (2020), studying the situation in the USA, find that older and more stable workers perceive a lower likelihood of losing their jobs.

Along the direct impacts of the pandemic, collateral effects concern the psychological consequences on the population (Crayne, 2020; Lu et al., 2021), which are unequally distributed within socio-economic groups and can unfortunately be detected months or years later (Jeong et al., 2016; Liu et al., 2012). Separation from friends and relatives, the loss of freedom and fear of contagions could induce dramatic psychological effects, and the potential benefits of a lockdown must be consequently weighed against the deriving costs (Luo et al., 2020; Salari et al., 2020; Vindegaard and Benros, 2020). The persistence of this effect is worrying and suggests that mitigation measures shall be organized as part of the lockdown planning process to minimize it.

Moreover, several studies (Alvarez and Hunt, 2005; Cukor et al., 2011) found a significant correlation between psychiatric history and the insurgence and persistence of post-traumatic psychological distress after quarantine and/or lockdown and require, therefore, specific attention.

Following the lesser of two evils principle, lockdown is necessary to be used as a counterpoison when the widespread diffusion of a virus must be slowed down. In this respect, all the possible measures must be taken by officials to reduce the collateral effects of isolation (Almulhim and Barahona, 2021). One way to reach this goal is to explain as clearly and completely as possible what is happening, why, how long it will last, provide activities to perform during isolation, ensuring basic supplies and reinforce the sense of altruism. This goal can be accomplished by the Central State Government as well as by other institutions such as universities.

Considering Italy specifically, the remarkable diffusion of the virus during the first months of 2020 brought to a general lockdown, sealing off the northern regions first, followed by the rest of the country. At the first stages of the process, positive attitudes toward lockdown measures could be connected to higher well-being and lower mental health symptoms (Prati, 2021).

Also, higher education levels seem to be correlated with higher levels of anxiety, which makes the choice of university students as population target for our analysis particularly interesting. All these results are confirmed by a study conducted in Rome and performed on individuals aged 18 years and over living in Italy (Mazza et al., 2020). This specific study shows, in particular, that a greater share of people is currently affected by high levels of anxiety, compared to the pre-pandemic average measured by European epidemiological statistics (Jacobi et al., 2014; Wittchen et al., 2011). This increase in anxiety level is in line with the main literature (see Brooks et al., 2020 for a review) and could be interpreted as COVID-19-related. Moreover, the medical history of mental health problems influences more pronounced levels of anxiety (Qiu et al., 2020; Wang et al., 2020). This result is motivated by the higher probability of re-emergence of past symptoms of psychological distress during periods of uncertainty such as pandemic outbreaks. Moreover, individuals affected by medical problems could perceive their health to be poor and potentially more exposed to new possibilities of contagion. The dependence of anxiety levels from individuals’ mental health history is one of the reasons why we decided to use a psychological anxiety test, which returns both a stable pre-pandemic measure of anxiety and a temporary measure referred to the current moment. Indeed, anxiety can be distinguished into the one connected to a personal trait and the one representing an emotional state (Cattell, 1966). The STAI-Y (State-Trait Anxiety Inventory) test (Spielberger, 1983), included in the survey, is a self-report test that provides two measures of anxiety: the State Anxiety Scale (S-Anxiety), which evaluates the current state of anxiety with items referring to how the people feel “right now”; and the Trait Anxiety Scale (T‐Anxiety), which refers to a stable measure of anxiety.

Following some recent studies on China and Italy (Amerio et al., 2021; Ho et al., 2020; Wang et al., 2020), gender significantly affects the impact of COVID-19 in terms of psychological health and, in particular, on anxiety, with stronger consequences for females than for males. This result is explained with a stronger vulnerability of females to stress, who more frequently develop post-traumatic distress.

Finally, several studies (see Mazza et al., 2020 for a review) showed that most of the increase in anxiety tends to be related to the policy measures put in place by the Governments and to the way people adapt to the pandemic crisis. The availability of data on the level of anxiety measured in two different moments lets us consider even this aspect.

## Data and methods

For our analyses, we use data from two *ad hoc* questionnaires, administered to the students of three Italian universities (Messina, Udine, and the Marche Polytechnic University) during the 2020 lockdown and one year after by means of the EUSurvey platform. The gathered evidence concerns demographic, economic, labor, context-based, on-line teaching, time use, and psychological well-being features.

All the students from all departments of the three universities were invited to take part in the first survey (Busetta et al., 2021), which was open from the 29th of April 2020 to the 17th of May 2020.

Then, starting from November 2020, Italy has been divided into three “colored” zones (red, orange, yellow), characterized by different restrictive measures on the basis of the severity of the spread of COVID-19 at the regional level (Panarello and Tassinari, 2022; Pelagatti and Maranzano, 2021). This decision made our choice of administering the surveys to students from three different areas in the country particularly appropriate concerning the administering of the second survey. This was held from the 9th of March 2021 to the 21st of April 2021, after the anti-COVID-19 vaccination campaign – which took place since December 2020 – and the third wave of COVID-19 in Italy, occurred since February 2021.

Since the 1st of September, 2021, university students started to be allowed to access university and specific economic activities only if in possession of the EU Digital COVID Certificate, which had not yet been announced in the period of our second survey.

In order to align with the EU General Data Protection Regulation (GDPR), the protocol of the survey was approved by the ethical committees of each of the three Universities. Detailed information about the aims of the study, composition of the research team – which includes a psychologist – and personal data transparency rules were provided to the prospective participants by means of an introductive section that respondents had to approve before accessing the survey questions. Each of the two surveys was completely anonymous; however, we asked respondents to provide a personal passcode, composed of the initial letter of their first names, the initial letter of their surnames, and the last four digits of their mobile phone numbers.

In total, 4,379 students had replied to the first survey, while we collected 3,580 completed questionnaires from the second wave. In this study, we only consider the students who provided their answers to both surveys, matching the observations using the passcode as key variable in order to obtain a balanced panel dataset. The final sample is composed of 317 students, observed in both waves.

The present paper focuses on anxiety, which is commonly defined as an adaptive emotion, preparing individuals to identify and face threats in order to guarantee their own survival.

As mentioned in the previous Section, we computed state and trait anxiety scores for each respondent. The State-Trait Anxiety Inventory (STAI) is composed of 40 items: 20 items for each subscale. Each subscale consists of 20 items that are rated on a 4-point Likert scale. The S-Anxiety items refer to the respondents’ feelings “at this moment”: (1) not at all, (2) somewhat, (3) moderately so, and (4) very much so. The T‐Anxiety items evaluate the frequency of feelings “in general”: (1) almost never, (2) sometimes, (3) often, and (4) almost always. For each subscale, specific instructions are provided. Each subtest has a range of scores from 20 to 80, the higher score indicating a higher level of anxiety. With a view to detecting clinically significant symptoms, anxiety can be classified into three different levels: low (from 20 to 37), moderate (from 38 to 44), and high (45 to 80).

We administered the STAI-Y test to university students in order to separate state anxiety (i.e., the one concerning the two pandemic periods, measured at a year’s distance) and trait anxiety (the level of anxiety as a personal characteristic).

The evaluation of structural breaks in the temporal dynamics of the analyzed phenomena and the analysis of the unobserved aspects of personality and well-being traits allow us to evaluate whether individual behaviors during the pandemic have changed compared to the habitual ones. The same test evaluating habitual and current anxiety levels has been administered in both survey waves.

Albeit the STAI is a validated test, we assessed the constructs’ internal consistency and reliability on our data, resulting in Cronbach’s alpha values of 0.9257 for Trait Anxiety, 0.9405 for State Anxiety at wave 1, and 0.9460 for State Anxiety at wave 2, which indicate an excellent level of consistency.

**Fig. 1:**
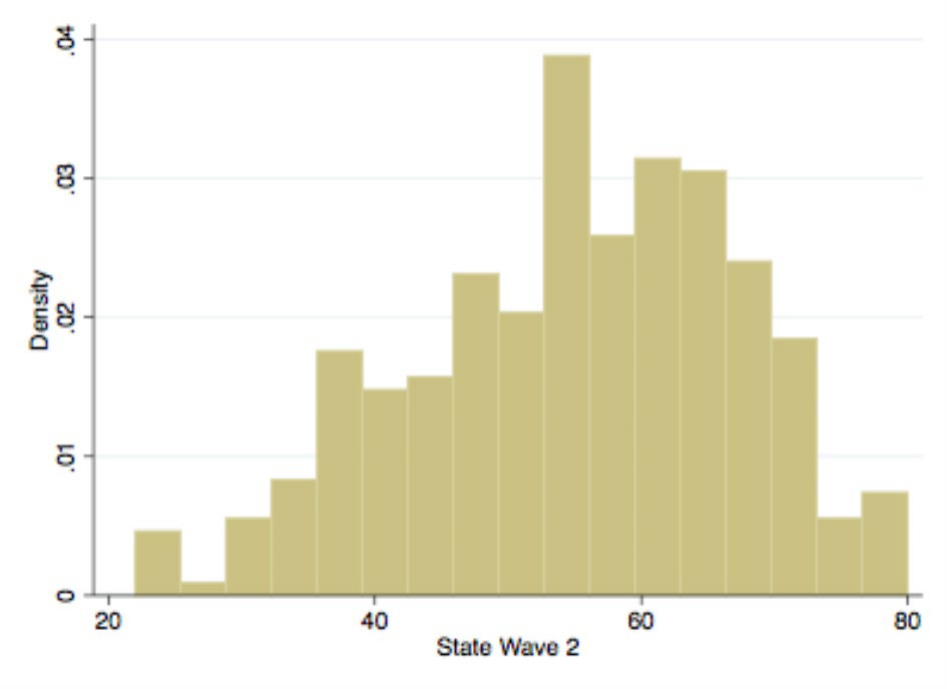
Distribution of State Anxiety level at wave 2

The State anxiety score collected in the second wave is used as dependent variable. We estimate a model in which State anxiety in 2020 and Trait anxiety in 2020 are used as regressors, together with information about demographic, economic and social characteristics. As the outcome variable is bounded above and below and its distribution is skewed to the left (see Fig. 1), it ought not to be modeled by means of a Normal-based regression model. Indeed, a logarithmic regression would be suitable for such a distribution. Therefore, we decided to regress a Poisson model, which provides better results than log-linear regressions (Wooldridge, 2010). A property of the Poisson regression model is that the mean and the variance must be equal. However, our data are over-dispersed (i.e., the variance is greater than the mean). To avoid modeling issues, we used the robust estimator of variance (Sandwich linearized estimator): in so doing, we obtain robust standard errors for the parameter estimates to control for mild violation of underlying assumptions (Cameron and Trivedi, 2009).

The independent variables used in our analysis are: the State anxiety score at wave 1 (continuous variable), the Trait anxiety score at wave 1 (continuous variable), gender (Woman: 1 = yes, 0 = otherwise), study cycle (Bachelor: 1 = yes, 0 = otherwise), study area (Medical: 1 = yes, 0 = otherwise), lessons attendance during the first semester, which goes from September 2020 to February 2021 (Lessons: 1 = yes, 0 = otherwise), the thought of whether their study path could be affected by the pandemic crisis (Study path expectations: 1 = Much worse than habitual, …, 5 = Much better than habitual), a dummy variable (COVID) that takes value 1 if the respondent, a friend or a relative had been infected by COVID-19 and 0 otherwise, and a dummy variable that takes value 1 if the student experienced drastic changes in his/her sleep pattern (Sleep). Moreover, we include a variable related to the change in the time spent on social networks during the pandemic compared to the previous period (Social Networks). This is built as the difference between the amount of time spent on social networks at the current time and the minutes the respondent habitually used to spend on such activities: positive values indicate an increase in the time dedicated to social networks compared to the habitual values. Finally, we consider the economic aspect. We include a variable stating whether at least one family member has lost his or her job (Job loss: 1 = yes, 0 = no) and a variable about the respondent’s expectations concerning the household’s financial/economic situation at the end of the pandemic. The latter was originally a 5-point Likert-scale variable and has been transformed into a new dichotomic variable to emphasize the impact of negative perceptions about the future (Low economic expectations: 1 = Much or somewhat worse than habitual, 0 = otherwise).

The total number of panel observations amounts to 317 (University of Udine 37%; Marche Polytechnic University 46%; University of Messina 19%), with the majority of students enrolled in a bachelor’s degree course (55%). The sample includes 223 females (70%) and 94 males (30%).

In the following table (Table 1) we show the descriptive statistics of the full sample, classified by level of trait anxiety at wave 1 (Low, from 20 to 37; Moderate, from 38 to 44; High, from 45 to 80).

**Table 1:** Characteristics of the survey respondents by STAI-Trait at wave 1

Level of Trait anxiety at wave 1	Low (obs. 80)		Moderate (obs. 73)		High (obs. 164)		Overall (obs. 317)	
Variable	Mean	Std. Dev.	Mean	Std. Dev.	Mean	Std. Dev.	Mean	Std. Dev.
STAI score: State wave 2	47.89	13.95	53.81	11.34	59.23	9.68	55.12	12.19
STAI score: State wave 1	46.31	13.59	48.92	11.78	56.45	9.52	52.15	12.05
STAI score: Trait wave 1							45.89	11.25
Low economic expectations	0.40		0.48		0.44		0.44	
COVID	0.51		0.58		0.49		0.51	
Job loss	0.14		0.14		0.17		0.15	
Woman	0.61		0.60		0.79		0.70	
Medical	0.20		0.22		0.17		0.19	
Bachelor	0.54		0.52		0.57		0.55	
Lessons	0.76		0.81		0.75		0.77	
Study path expectations								
*Much worse than habitual*	0.18		0.18		0.19		0.19	
*Somewhat worse than habitual*	0.31		0.34		0.41		0.37	
*About the same*	0.35		0.26		0.26		0.28	
*Somewhat better than habitual*	0.13		0.18		0.09		0.12	
*Much better than habitual*	0.03		0.04		0.05		0.04	
Sleep	0.16		0.23		0.25		0.22	
Social networks	0.10		0.29		0.35		0.27	

In general, our data confirm the robustness of the Trait Anxiety Scale, as students’ Trait Anxiety scores measured at one year’s distance do not show significant differences. Besides, our descriptive analysis shows an increase in State Anxiety scores between the two waves. Moreover, State Anxiety scores are higher than the Trait Anxiety one. In particular, most students present high levels of State anxiety at both waves (71% and 78%, respectively).

**Fig. 2:**
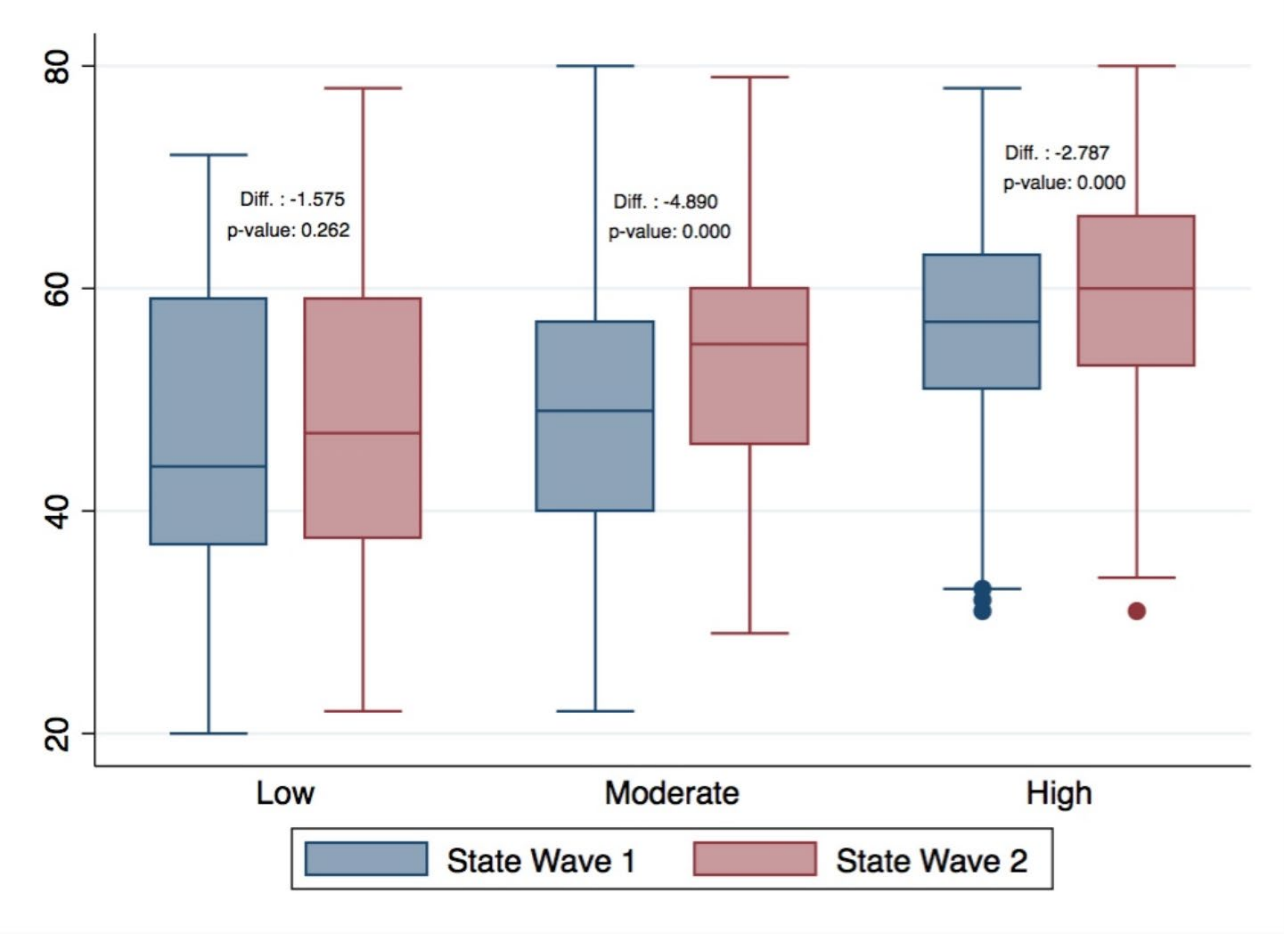
Level of State Anxiety at wave 1 and wave 2 by level of Trait Anxiety

We observe that 204 students reside in the “high anxiety” category at both waves (65%). Moreover, 44 students who had low or moderate anxiety levels at wave 1 have increased their level to “high” at wave 2. Only for 22 students, we observe a decrease in the level of anxiety from high to moderate or low. To better understand the differences in the development of state anxiety that can be observed depending on students’ level of anxiety pre-pandemic and at the beginning of it (H2), we show the State Anxiety levels at waves 1 and 2, by level of Trait Anxiety (Fig. 2).

These descriptive results are in line with our hypothesis (H1) that students’ average level of anxiety increased during the pandemic (State Anxiety) compared to the habitual level (Trait Anxiety) and that State Anxiety increased at one year’s distance.

In accordance with the literature on the topic (Czymara et al., 2021, Sucuoğlu, 2018), we find statistically significant differences between men and women, in both waves (H4). In particular, the State Anxiety level measured in 2020 was 47.5 and 54.1 for men and women, respectively (t = -4.6510, p-value = 0.0000), while in the second wave it was 50.8 and 56.9, respectively (t = -4.1826, p-value = 0.0000). As regards trait anxiety, we also find statistically significant differences (41.9 for men and 47.6 for women, t = -4.1961, p-value = 0.0000).

**Fig. 3:**
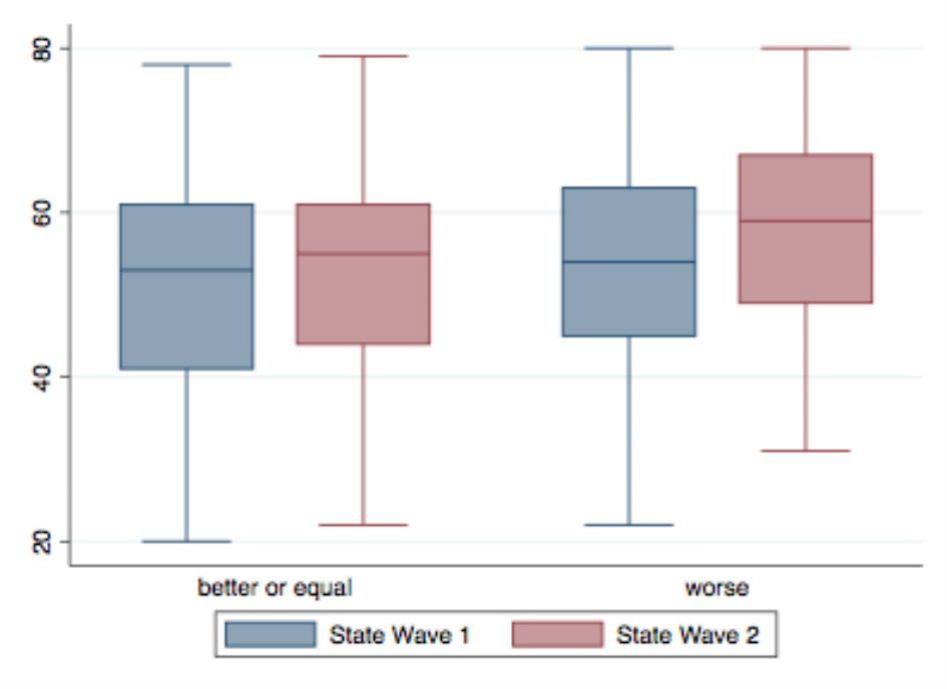
Level of State Anxiety at wave 1 and wave 2 by level of economic expectancy

Regarding expectations about the household’s economic/financial situation at the end of the pandemic, we find significant differences (t = -3.3129, p-value = 0.001) in State anxiety at wave 2 between students thinking that their situation is going to be better or equal than before the pandemic (53.1) and those thinking that it is going to be worse than usual (57.6). Moreover, we observe small differences in State anxiety between the two waves in between the subsample of “optimistic” students, while considerable increases in state anxiety levels are shown within the group of students who think that the household’s economic situation will be worse at the end of the pandemic (Fig. 3). This result supports our H3 hypothesis that students’ economic expectancies can affect their anxiety level.

## Results

Table 2 shows the results from our model.

**Table 2:** Poisson regression results (dependent variable: STAI-State at wave 2)

	Coef.	Robust Std. Err.	IRR	Robust Std. Err.	p-value
STAI score: State wave 1	0.008	0.001	1.008	0.001	***
STAI score: Trait wave 1	0.004	0.001	1.004	0.001	***
Low economic expectations	0.060	0.018	1.062	0.019	***
COVID	0.021	0.018	1.021	0.018	
Job loss	−0.009	0.024	0.991	0.024	
Woman	0.046	0.022	1.047	0.023	*
Medical	0.042	0.021	1.043	0.022	*
Bachelor	−0.064	0.040	0.938	0.037	
Lessons	−0.078	0.028	0.925	0.026	**
Bachelor*Lessons	0.121	0.045	1.128	0.051	**
Study path expectations					
*Somewhat worse than habitual*	−0.085	0.026	0.919	0.024	**
*About the same*	−0.090	0.029	0.913	0.027	**
*Somewhat better than habitual*	−0.109	0.037	0.897	0.033	**
*Much better than habitual*	−0.102	0.064	0.903	0.058	
Sleep	0.069	0.020	1.072	0.022	***
Social Networks	0.039	0.011	1.040	0.011	***
Constant	3.432	0.069	30.934	2.125	***

Note: * p<0.05; ** p<0.01; *** p<0.001

The coefficient for trait anxiety is positive and highly significant, meaning that people who are tendentially more anxious are more likely to report higher levels of state anxiety at the current time. The coefficient for state anxiety score at wave 1 is positive and highly significant, implying that a higher anxiety level at the beginning of the pandemic predicts a higher increase in anxiety after one year of COVID-19-related restrictions, compared to individuals characterized by lower levels of anxiety in April 2020. Increasing levels of anxiety for individuals who were already characterized by high anxiety scores are particularly dangerous, as suicide risk in individuals with anxiety disorders tends to be higher compared to that of individuals with a moderate level of anxiety (Khan et al., 2002).

People who believe that their household’s financial situation will be worse than that usually experienced before the pandemic are more likely to exhibit a higher level of state anxiety.

By contrast, unsuspiciously, other important features such as having had family members infected by COVID-19 and/or having experienced a short-term loss in their income, due to a job loss suffered by one of the family members, is not going to significantly affect students’ anxiety. This result is impressive, as it means that facing the disease and/or its undergone economic consequences does not have a significant impact on anxiety, while uncertainty about the future economic conditions of the household has, in line with how hypothesized by Godinic et al. (2020).

Women, compared to men, are more likely to experience higher levels of anxiety after one year of pandemic, keeping the previous levels of anxiety fixed.

University students from medical areas are more likely to experience a higher level of anxiety, due to being more informed about the way the disease works and its connected consequences.

Our results do not show any relevant difference in terms of anxiety between graduate and postgraduate (master’s, PhD, etc.) students.

In general, students who attended university courses during the current year, even if sometimes at distance, are more likely to report lower levels of anxiety. However, when focusing on the interaction of bachelor’s students who attended university lessons, we derive that this category is more likely to show a high anxiety level, most probably because they may already be stressed by the school-to-college transition and the inclusion of distance lessons in their daily routines may produce an increase in their anxiety level.

As we are focusing on university students, we included expectations about the study path in our model. High levels of anxiety are shown to be produced by the thought of a significantly negative effect of the pandemic on their university path.

Moreover, a drastic change in sleep routine (sleeping either less or more compared to the habitual levels) is positively correlated with anxiety.

Our data show that students’ frequency in the use of social networks is higher than in the pre-pandemic period. Most probably, this is a reaction against the strong reduction in vis-a-vis contacts imposed by the COVID-19 restrictions (Gioia et al., 2021). Such a palliative for non-virtual contacts with peers produces negative effects on psychological well-being, as it is shown to be positively correlated with anxiety levels.

In the early stage of the pandemic, a huge increase in texting, social media and video conference activity was detected (Richter, 2020). For this reason, we also used the level and quality of students’ internet connection and the availability of these technologies as a factor facilitating their capacity of adaptation to social restrictions, able to reduce, in this way, the impact in terms of increasing anxiety.

## Discussion and conclusions

Italy has chosen to manage the pandemic by imposing a strict lockdown at the beginning, then adopting a strategy of alternating closures and reopenings in order to keep the number of infections below an alert threshold, generating an atmosphere of general uncertainty and ambiguity among the population, in terms of reopening schedules, short-term economic prospects, financial responses by the government, and speed of recovery from the health crisis. Indeed, these aspects could have induced a reassessment of students’ expectations about the future. Other ways of dealing with the pandemic could have been adopted: differently from Italy, countries such as Germany, the United Kingdom, and Australia opted for a long-term strategy aimed at guaranteeing a lower level of uncertainty. On the one hand, these countries maintained a huge degree of restrictions for several months before finally relaxing the containment measures for the long haul; on the other, they provided certain and rapid wage compensations for those directly affected by the closures.

As we think that the Italian strategy could imply deep consequences both at the psychological and economic levels, we decided to study how students’ anxiety level changes over the course of the COVID-19 crisis, as a result of the collateral insecurity effects induced by the pandemic. Considering that different people have different perceptions about their future financial situation (Panarello, 2021), their level of anxiety can vary under equivalent conditions.

To do so, we performed a Poisson regression model in which students’ State Anxiety level measured one year after the onset of the pandemic is explained by gender, economic expectancies, job losses, expected consequences on their university path, undergone changes in daily routines (sleep, social networks), proximity to COVID-19 (having had a family member infected by the disease), medical knowledge (proxied by field of study), lessons’ attendance, study cycle.

Our results show that anxiety has hugely worsened since the beginning of the pandemic and during the course of it, especially for women. State anxiety underwent a higher increase for individuals who were already characterized by a high level of state anxiety at the beginning of the pandemic and who originally had a high level of trait anxiety. This result is particularly dangerous because high levels of anxiety are usually associated with social isolation and loneliness, which, in turn, are connected with increased suicide risk (e.g., Calati et al., 2019, Van Orden et al., 2010). In support of the harmfulness of this element, French President Emmanuel Macron declared, on April 14th, 2021: “We are seeing the rise of something that we did not experience during the first confinement, an anxiety and anguish among the youngest people, which is reflected in the figures”^1^. For such a reason, he promised free psychological counseling during the COVID-19 crisis for children aged between 3 and 17. Our results, as the chosen universities are located in the three Italian macroareas (South, Center, and North), confirm the need to extend such policy measure to Italian university students, even by making use of distance tools such as telemedicine (Drago et al., 2021; Romani et al., 2021). Moreover, particular attention must be paid to the most vulnerable categories, e.g., by providing gender-specific health-care policies (Gatto et al., 2022; Koch and Park, 2022). In particular, we believe that policymakers should act in order to avoid the resulting increase in anxiety to translate into an amplified suicide risk, as predicted by the literature (Reger et al., 2020, Tedeschi and Calhoun, 2004).

Following our results, better economic expectancies are associated with lower anxiety levels: thus, we confirm the crucial role of economic insecurity on psychological well-being (Rohde et al., 2016). Hence, focusing on economic expectancies should become a priority in the current policymaking agenda, with a view to reducing people’s anxiety levels during the post-pandemic recovery. Moreover, in order to manage the spread of COVID-19, it is crucial to enhance the society’s trust in the healthcare system (Antinyan et al., 2021).

More into detail, our outcomes show that other factors may affect the level of university students’ state anxiety in 2021: pursuing of a medical degree, lessons’ attendance, expected consequences of the pandemic on university path, and differences in sleep routine and social networks’ usage. Conversely, the anxiety growth seems not to be influenced by job loss, having had a family member infected by COVID-19, and study cycle.

The dynamics of the onset and development of the COVID-19 pandemic in Italy also reflect the Italian territorial differences, with most deaths concentrated in the north, the most industrialized and polluted area (Aloisi et al., 2022; Bosa et al., 2021). Moreover, the recent development of COVID-19 vaccinations, especially in Europe and the USA as the most advanced areas in the pharmaceutical sector (Aldieri et al., 2020), is also expected to increase anxiety levels in the near future (Bodner et al., 2022). Therefore, in an upcoming stream of research, it would be interesting to collect data from further universities, with a view to assessing the long-term effects of the pandemic considering the different pandemic strength at the local and regional level and the effects brought about by vaccinations.

## Footnotes

1
https://www.euronews.com/2021/04/15/french-president-macron-pledges-free-counselling-for-students-during-covid-pandemic.

## Declaration

### Availability of data and material

Data shall be made available upon request.

### Code availability

Code shall be made available upon request.
